# Downregulation of histone methyltransferase *SET8* inhibits progression of hepatocellular carcinoma

**DOI:** 10.1038/s41598-020-61402-7

**Published:** 2020-03-11

**Authors:** Jianhua Wu, Kuangyuan Qiao, Yanming Du, Xiaoyun Zhang, Haichao Cheng, Li Peng, Zhanjun Guo

**Affiliations:** 1 Animal center, The Fourth Hospital of Hebei Medical University, Shijiazhuang, P.R. China; 2 Department of Rheumatology and Immuology, The Fourth Hospital of Hebei Medical University, Shijiazhuang, P.R. China; 3 Department of Hepatobiliary Surgery, The Fourth Hospital of Hebei Medical University, Shijiazhuang, P.R. China; 4 Basic Medical College, Hebei Medical University, Shijiazhuang, P.R. China

**Keywords:** Gastrointestinal cancer, Tumour biomarkers

## Abstract

The expression of lysine methyltransferase SET8, which is involved in carcinogenesis of many types of human cancers through monomethylation of histone H4 lysine 20 (H4K20), is associated with the prognosis of hepatocellular carcinoma (HCC). We performed a functional analysis for SET8 to assess its effect on HCC progression. SET8 knockdown inhibited proliferation, migration and invasion of HCC cells. SET8 knockdown also inhibited tumour growth in a human xenograft mouse model. Overexpression of SET8 displayed the reverse effect, while treatment with the SET8 inhibitor UNC0379 produced an effect similar to SET8 knockdown. In addition, drug sensitivity testing in *SET8-siRNA* transfected HCC cells indicated that docetaxel inhibited cell growth dramatically, as demonstrated by the Cell Counting Kit-8 (CCK-8) assay. Furthermore, gene expression microarray analysis showed that genes altered after SET8 knockdown were clustered in pathways related to tumorigenesis and metastasis. Our data suggests that targeting SET8 for HCC therapy can inhibit the proliferation and invasion of HCC cells as well as increase their sensitivity to chemotherapy.

## Introduction

Hepatocellular carcinoma (HCC) is a highly aggressive malignant tumour that accounted for approximately 782,500 new liver cancer cases and 745,500 deaths worldwide in 2012^1^. Despite significant developments in the diagnosis and treatment of HCC, the overall survival of patients with HCC remains low due to distant metastasis, local recurrence, treatment resistance, and lack of early diagnosis. Molecular targets underlying the mechanisms of HCC development, progression and metastasis may provide novel effective therapeutic targets for HCC treatment^2^.

Post-translational histone modifications, including methylation, acetylation, phosphorylation, ubiquitination, and ADP-ribosylation, are involved in a number of cellular processes such as mitosis, meiosis, DNA replication, and the DNA damage response^3^. Histone H4 methylation is one of the most intriguing histone modifications with the majority of histone H4 methylation occurring in the N-terminal tail of lysine 20 (H4K20). *PR-Set7/Set8/KMT5a* (*SET8*) encodes an H4K20 monomethyltransferase that is implicated in normal cell cycle progression including DNA replication, DNA damage repair, and cell cycle control^4,5^. SET8 could exert specific effects at the origin of DNA replication through interacting with the DNA replication factor proliferating cell nuclear antigen (PCNA), and SET8 depletion causes cells to arrest in the S phase with increased DNA damage and apoptosis^6–10^. Additionally, SET8 binds and methylates non-histone oncoproteins such as p53, TWIST, Wnt, estrogen receptor alpha (ERα) and androgen receptor (AR), and recruits p53BP1 during the DNA double-strands break response^11–14^. Furthermore, SET8 mediates the dual transcriptional activities of TWIST and promotes epithelial-mesenchymal transition leading to increased metastatic capacity of breast cancer cells^12,15^.

We have previously identified that altered SET8 expression modified HCC outcome^16^. Here, we performed a functional analysis for SET8 to assess its effect on HCC progression.

## Methods

### Cell culture and transfection

Six human hepatic carcinoma cell lines (QGY-7701, Hepg2, LM3, Huh-7, SMMC-7721, Hep3B) were purchased from the Shanghai Institute of Biological Sciences, Chinese Academy of Sciences (Shanghai, China) and cultured in DMEM/RPMI-1640 medium (Gibco^TM^ Life Technologies, Grand Island, NY) supplemented with 10% foetal bovine serum (FBS) (Gibco^TM^ Life Technologies, Grand Island, NY) in a humidified incubator containing 5% CO_2_ at 37 °C.

SMMC-7721 cells in the logarithmic growth phase were seeded into 6-well plates. *psi-H1*-*SET8siRNA* and *psi-H1* plasmids expressing green fluorescent protein (GFP) (GeneCopoeia, Rockville, MD) were transfected into SMMC-7721 cells using Lipofactamine 2000 (Invitrogen, San Diego, CA). To overexpress SET8, the coding sequence of SET8 was amplified and subcloned into the pEZ-M61 vector, having a puromycin selection marker (GeneCopoeia). Huh-7 cells were then transfected with *SET8*-pEZ-M61 and pEZ-M61 plasmids using lipofectamine 2000 (Invitrogen). After 72 h of incubation, 0.6 µg/ml puromycin (Sigma-Aldrich, St. Louis, MO) was added into each well for 10–12 days and resistant clones were selected. The siRNA and cDNA clone sequences are listed in Table 1.

**Table 1:** The sequence of siRNA and cDNA clone of SET8.

Name	Sequence
SET8-siRNA1	CAGAATCGCAAACTTACGGAT
SET8-siRNA2	GAATGAAGATTGACCTCATCG
SET8-siRNA3	GCCTAGGAAGACTGATCAATC
SET8-siRNA4	GGCGCTCACTGAAGTGTATGA
ORF nucleotide sequence of SET8	ATGGCTAGAGGCAGGAAGATGTCCAAGCCCCGCGCGGTGGAGGCGGCGGCGGCGGCGGCGGCGGTGGCAGCGACGGCCCCGGGCCCGGAGATGGTGGAGCGGAGGGGCCCGGGGAGGCCCCGCACCGACGGGGAGAACGTATTTACCGGGCAGTCAAAGATCTATTCCTACATGAGCCCGAACAAATGCTCTGGAATGCGTTTCCCCCTTCAGGAAGAGAACTCAGTTACACATCACGAAGTCAAATGCCAGGGGAAACCATTAGCCGGAATCTACAGGAAACGAGAAGAGAAAAGAAATGCTGGGAACGCAGTACGGAGCGCCATGAAGTCCGAGGAACAGAAGATCAAAGACGCCAGGAAAGGTCCCCTGGTACCTTTTCCAAACCAAAAATCTGAAGCAGCAGAACCTCCAAAAACTCCACCCTCATCTTGTGATTCCACCAATGCAGCCATCGCCAAGCAAGCCCTGAAAAAGCCCATCAAGGGCAAACAGGCCCCCCGAAAAAAAGCTCAAGGAAAAACGCAACAGAATCGCAAACTTACGGATTTCTACCCTGTCCGAAGGAGCTCCAGGAAGAGCAAAGCCGAGCTGCAGTCTGAAGAAAGGAAAAGAATAGATGAATTGATTGAAAGTGGGAAGGAAGAAGGAATGAAGATTGACCTCATCGATGGCAAAGGCAGGGGTGTGATTGCCACCAAGCAGTTCTCCCGGGGTGACTTTGTGGTGGAATACCACGGGGACCTCATCGAGATCACCGACGCCAAGAAACGGGAGGCTCTGTACGCACAGGACCCTTCCACGGGCTGCTACATGTACTATTTTCAGTATCTGAGCAAAACCTACTGCGTGGATGCAACTAGAGAGACAAATCGCCTAGGAAGACTGATCAATCACAGCAAATGTGGGAACTGCCAAACCAAACTGCACGACATCGACGGCGTACCTCACCTCATCCTCATCGCCTCCCGAGACATCGCGGCTGGGGAGGAGCTCCTGTATGACTATGGGGACCGCAGCAAGGCTTCCATTGAAGCCCACCCGTGGCTGAAGCATTAG

### Western blot analysis

Total protein was extracted using radio immunoprecipitation assay (RIPA) lysis buffer with freshly added protease inhibitor (Roche, Basel, Switzerland). Western blot analysis was performed as described previously^17^. Briefly, 40 μg total protein extract was loaded into a 10% sodium dodecyl sulphate polyacrylamide gel (SDS-PAGE) followed by transfer onto a polyvinylidene difluoride (PVDF) membranes (Roche, Basel, CH). Membranes were blocked with 5% skim milk, incubated first with rabbit polyclonal antibodies against human SET8 (1:1000; Abcam, Cambridge, UK), p53 (1:2,000; Santa Cruz, CA), anti-p53K382me1(1:1000; Affinity Biosciences, Cincinnati, OH)^11^, or β-actin (1:10,000; Santa Cruz, CA) overnight at 4 °C, followed by incubation with secondary HRP-conjugated anti-rabbit IgG antibody (1:5000; Thermo Fisher, New York, NY). Proteins were visualized with an enhanced chemiluminescence reagent (Thermo Fisher, New York, NY) using the FluorChem® HD2 protein imprinting imaging system (Alpha InnoTec, San Leandro, CA).

### Cell proliferation assay

Cell proliferation was examined using the Cell Counting Kit (CCK)−8 (Dojindo Lab, Kumamoto, Japan). Briefly, cells stably transfected with *psi-H1-SET8siRNA* or *SET8*-*pEZ-M61* in the logarithmic growth phase were seeded into 96-well microplates with a density of 1×10^3^ cells per well. Non-transfected SMMC-7721, Huh-7 and Hepg2 cells were also treated with the SET8 inhibitor UNC0379 (MCE, Monmouth Junction, NJ) for 72 h. At each time point (0, 12, 24, 48, 60 and 72 h), 10 µl of diluted CCK-8 was added into each well and cells were incubated in a 5% humidified CO_2_ incubator for 2 h at 37 °C. Absorbance was measured at 450 nm using a Microplate Autoreader (Bio-Tek; Instruments, VT).

### Wound healing assay

Cells were cultured in 6-well plates. A scratch was created on the cell layer using a 200 µl pipette tip when cells reached 100% confluence. Subsequently, cells were washed twice with PBS. Images of the cells in each group were captured at 0, 12, 24 and 48 h after scratching. At least five fields were analysed for each scratch and cell migration rates were calculated using the following formula^18^.

$${Percentage}\,{wound}\,{healing}=\frac{({\rm{wound}}\,{\rm{length}}\,{\rm{at}}\,0\,{\rm{h}})-({\rm{wound}}\,{\rm{length}}\,{\rm{at}}\,12,\,24{\rm{h}}\,{\rm{or}}\,48{\rm{h}})}{{\rm{wound}}\,{\rm{length}}\,{\rm{at}}\,0\,{\rm{h}}}\times 100 \% $$

Each experiment was repeated three times.

### Cell invasion assay

Cell invasion assays were performed using 8 μm Transwell chambers containing polycarbonate filters (pore size, 8; Corning, NY) with Matrigel (BD Biosciences, NJ). Cells stably transfected with *psi-H1-SET8siRNA* or *SET8*-*pEZ-M61* (5×10^4^ cells per well) grown in 200 μl serum-free medium were placed in the upper chambers; SMMC-7721 and Huh-7 cells treated with UNC0379 in 200 μl serum-free medium were also placed in the upper chambers. 500 μl DMEM medium containing 15% FBS was added to the lower chamber, and cells were allowed to invade through the matrigel for 24 h at 37 °C. Cells which invaded into the underside of the membrane were fixed with 4% paraformaldehyde and stained with 0.1% crystal violet. Stained cells were counted under an inverted microscope (Nikon, Tokyo, Japan) in five randomly selected fields (magnification, x 200).

### Flow cytometry

Cell apoptosis was assessed by flow cytometry analysis. SMMC-7721 cells were harvested and washed twice with cold PBS and once with binding buffer, and then incubated with PE-conjugated Annexin V and 7-AAD (BD Biosciences, Pharmingen, San Diego, CA) for 15 minutes in the dark at 25 °C. Cells were then resuspended in binding buffer and analyzed using a fluorescence-activated cell sorting (FACS) Aria II flow cytometer (BD Biosciences). Each experiment was repeated three times.

### Nude mice for tumour cell xenograft assays

Twenty-four four-week-old female *BALB/c* nude mice were purchased from Charles River Laboratories [Beijing, China; permission no. SCXK (Jing) 2016–0006]. The mice were kept in a clean environment at the Laboratory Animal Centre in the Fourth Hospital of Hebei Medical University and provided standard rodent chow and water *ad libitum*. Animal experiments were conducted in accordance with the National Institutes of Health (USA) guidelines for the care and use of laboratory animals^19^ and approved by the Institutional Animal Care and Ethics Committee at The Fourth Hospital of Hebei Medical University.

SMMC-7721 cells (5×10^6^ cells/mouse in 200 µl DMEM) stably transfected with *psi-H1-SET8siRNA* or *psi-H1* were subcutaneously injected in the left scapular region of each mouse. Tumour growth was monitored at regular intervals by measuring tumour diameter using a calliper. Tumour volume was determined using the following formula.

$${\rm{Tumour}}\,{\rm{size}}=\frac{{\rm{length}}\times {{\rm{width}}}^{2}}{2}$$

*In vivo* green fluorescent imaging was performed using the NightOwl Bioimager (Berthold Technologies, Bad Wildbad, Germany) once a week. Signal intensity was quantified using the WinLight32 software package (Berthold Technologies).

### Chemosensitivity assay

Drug sensitivity testing for the chemotherapy reagents cisplatin, docetaxel, 5-fluorouracil, and etoposide (TCI chemicals, Shanghai, China) was performed in *SET8-siRNA* transfected SMMC-7721 cells with the CCK-8 assay as described above. Cells were treated for 72 h prior to CKK-8 assay with the following: cisplatin, 0, 0.2, 0.4, 0.6, 0.8, 1.0, 1.2 mg/ml; docetaxel, 0, 20, 40, 60, 80, 100, 120 mg/ml; 5-fluorouracil (5-Fu), 0, 0.3, 0.6, 0.9, 1.2, 1.5, 1.8 mg/ml; etoposide, 0, 0.2, 0.4, 0.6, 0.8, 1.0, 1.2 mg/ml.

### Microarray analysis

The microarray experiment was commissioned to CapitalBio Corporation (Beijing, China). Briefly, SMMC-7721 cells stably transfected with *psi-H1-SET8siRNA* or *psi-H1* were harvested and washed three times with PBS. Total RNA was extracted using TRIzol (Invitrogen, Carlsbad, CA). Subsequently, RNA samples were reverse-transcribed to generate cDNA. Three biological replicates for each group were used. Microarray manufacturing and processing were performed as described^20^. Microarrays were scanned on a GeneChip Scanner 3000 (Affymetrix, Santa Clara, CA), and data analysis was performed using GeneChip Operating Software (GCOS version 1.4, Affymetrix, Santa Clara, CA). Significantly altered genes following SET8 knockdown were investigated for biological processes and signalling pathways using the cytoscape plug-in of Reactome FI^21^.

### Statistical analysis

Data are expressed as means ± standard deviation. Statistical analysis between two groups was performed with Student’s *t* test, and the comparison between three or more groups was performed with One-way ANOVA. P < 0.05 was considered statistically significant. Statistical analyses were performed using SPSS 19.0 software package (SPSS Inc., Chicago, IL).

## Results

### Downregulation of SET8 inhibits proliferation, migration and invasion of HCC cells

**Figure 1:**
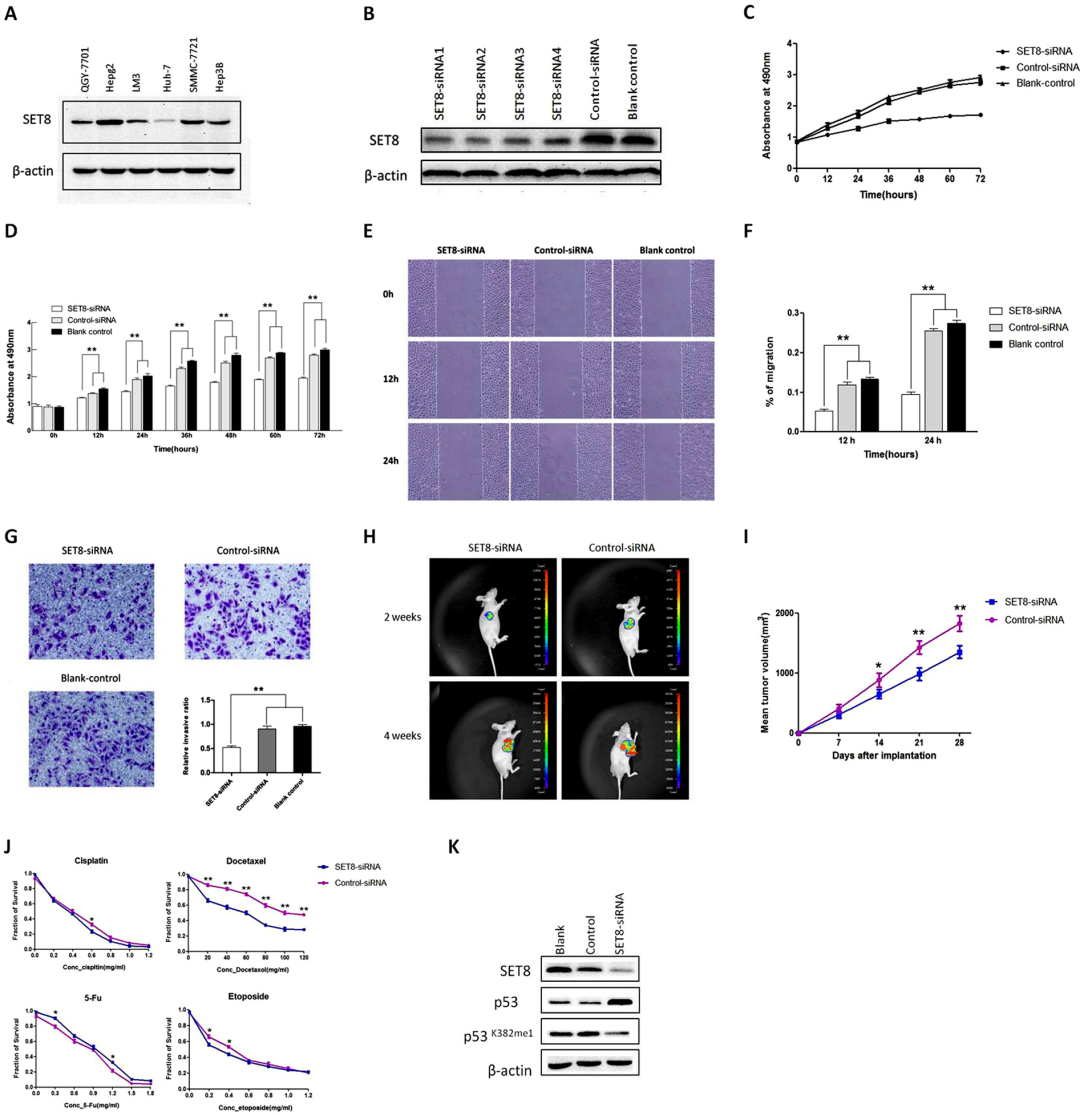
SET8 knockdown inhibits proliferation, migration and invasion of HCC cells and inhibits HCC cell growth *in vivo*. (**A**) Expression of SET8 protein levels was examined in different HCC cell lines with Western blotting. (**B**) Expression of SET8 protein was examined with Western blotting in SMMC-7721 cells transfected with *psi-H1-SET8* siRNA or *psi-H1* plasmids. (**C**) CCK-8 assay showing that SET8 knockdown inhibits proliferation of SMMC-7721 cells. (**D**) Quantification of results from C. (**E**) Wound healing assay showing that SET8 knockdown suppresses migration of SMMC-7721 cells. (**F**) Quantification of results from E. (**G**) Transwell assay showing that SET8 knockdown suppresses invasion of SMMC-7721 cells. Effects of SET8 knockdown on HCC cell growth *in vivo*. (**H**) The tumour tissues were observed using an animal tissue imaging system. (**I**) Growth curves of xenograft tumours. (**J**) Effects of SET8 knockdown on chemotherapy sensitivity after treatment with cisplatin, docetaxel, etoposide, or 5-Fu in SMMC-7721 cells. Cells transfected with either *SET8-siRNA* or control-siRNA were treated with increasing concentrations of cisplatin, docetaxel, etoposide, or 5-Fu. CCK-8 assay showing cell survival. (**K**) Western blot showing the protein levels of SET8, p53, and p53K382me1 upon treatment with the indicated siRNAs in SMMC-7721 cells. **p < 0.01, *p < 0.05.

SET8 protein expression was assayed in HCC cell lines including QGY-7701, Hepg2, LM3, Huh-7, SMMC-7721 and Hep3B cells with Western blotting (Fig. 1A). Since SMMC-7721 displayed high SET8 expression whereas Huh-7 displayed low SET8 expression, we use these two cell lines for subsequent knockdown and overexpression assays. Four psi-H1-*SET8siRNAs* were successfully transfected into SMMC-7721 cells. Western blot analysis indicated that SET8 levels significantly decreased in *SET8* siRNA2 transfected cells compared with other *SET8* siRNAs transfected cells (Fig. 1B); therefore, we used the *SET8* siRNA2 construct for subsequent analysis.

CCK-8 assays were performed to determine the proliferation ability of SMMC-7721 cells. SET8 knockdown markedly inhibited HCC cell proliferation from 12 to 72 h compared to *psi-H1*-transfected cells and blank control cells (P < 0.01, Fig. 1C,D). Migration was assessed with the wound healing assay. Compared with SMMC-7721 cells transfected with control-siRNA and blank control cells, the migration ratio was obviously decreased in SMMC-7721 cells transfected with *SET8-*siRNA at 12 and 24 h (P < 0.01, Fig. 1E,F). In transwell assay, SET8 knockdown also dramatically inhibited SMMC-7721 cell invasion (P < 0.01, Fig. 1G). To further investigate the mechanism of cell proliferation inhibition by SET8, apoptosis was measured by Annexin V-PE/7-AAD double staining with flow cytometry. No significant difference in apoptosis was found between *SET8-siRNA*, control siRNA and blank control groups (data not shown). These finding suggests that SET8 knockdown inhibited proliferation, migration and invasion of SMMC-7721 cells.

While p53 expression increased, monomethylation of p53 K382 (p53K382me1) decreased upon SET8 silencing (Fig. 1K). These data demonstrated that SET8 could modulate p53 expression through methylation of K382 in HCC cells.

To further evaluate the *in vivo* effect of SET8 knockdown on tumour growth, the growth rate of SMMC-7721 xenografts stably transfected *SET8-siRNA* was compared to xenografts transfected with control-siRNA. As shown in Fig. 1H,I, the growth of *SET8-siRNA* xenografts was significantly decreased compared with that of control-siRNA xenografts, as assessed with green fluorescent imaging. The tumour volume of *SET8-siRNA* xenografts was smaller than that of control-siRNA xenografts at 14, 21 and 28 days after implantation (P < 0.05) according to calliper measurement. These results suggest that SET8 knockdown inhibited growth of SMMC-7721 cells *in vivo*.

### Overexpression of SET8 promotes proliferation, migration and invasion of HCC cells

**Figure 2:**
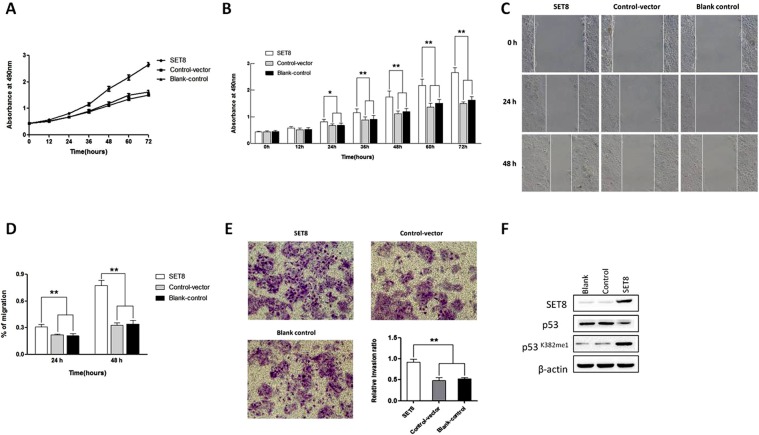
SET8 overexpression promotes proliferation, migration, and invasion of HCC cells. (**A**) CCK-8 assay showing that SET8 overexpression promotes proliferation of Huh-7 cells. (**B**) Quantification of results from A. (**C**) Wound healing assay showing that SET8 overexpression promotes migration of Huh-7 cells. (**D**) Quantification of results from C. (**E**) Transwell assay showing that SET8 overexpression stimulates invasion of Huh-7 cells. (**F**) Western blot showing the protein levels of SET8, p53 and p53K382me1 upon SET8 overexpression in Huh-7 cells. **p < 0.01, *p < 0.05.

The effect of SET8 overexpression on HCC progression in terms of proliferation, migration and invasion was evaluated in Huh-7 cells. Cells transfected with *SET8-pEZ-M61* promoted proliferation from 24 to 72 h (P < 0.05, Fig. 2A,B), migration (P < 0.05, Fig. 2C,D) at 24 and 48 h), and invasion (P < 0.01, Fig. 2E) compared with cells transfected with *pEZ-M61* or blank control cells. SET8 overexpression not only reduced p53 expression, but also increased p53K382mel (Fig. 2F). These data demonstrated that overexpression of SET8 promoted proliferation, migration and invasion of HCC cells.

### An inhibitor of SET8 inhibits proliferation, migration and invasion of HCC cells

**Figure 3:**
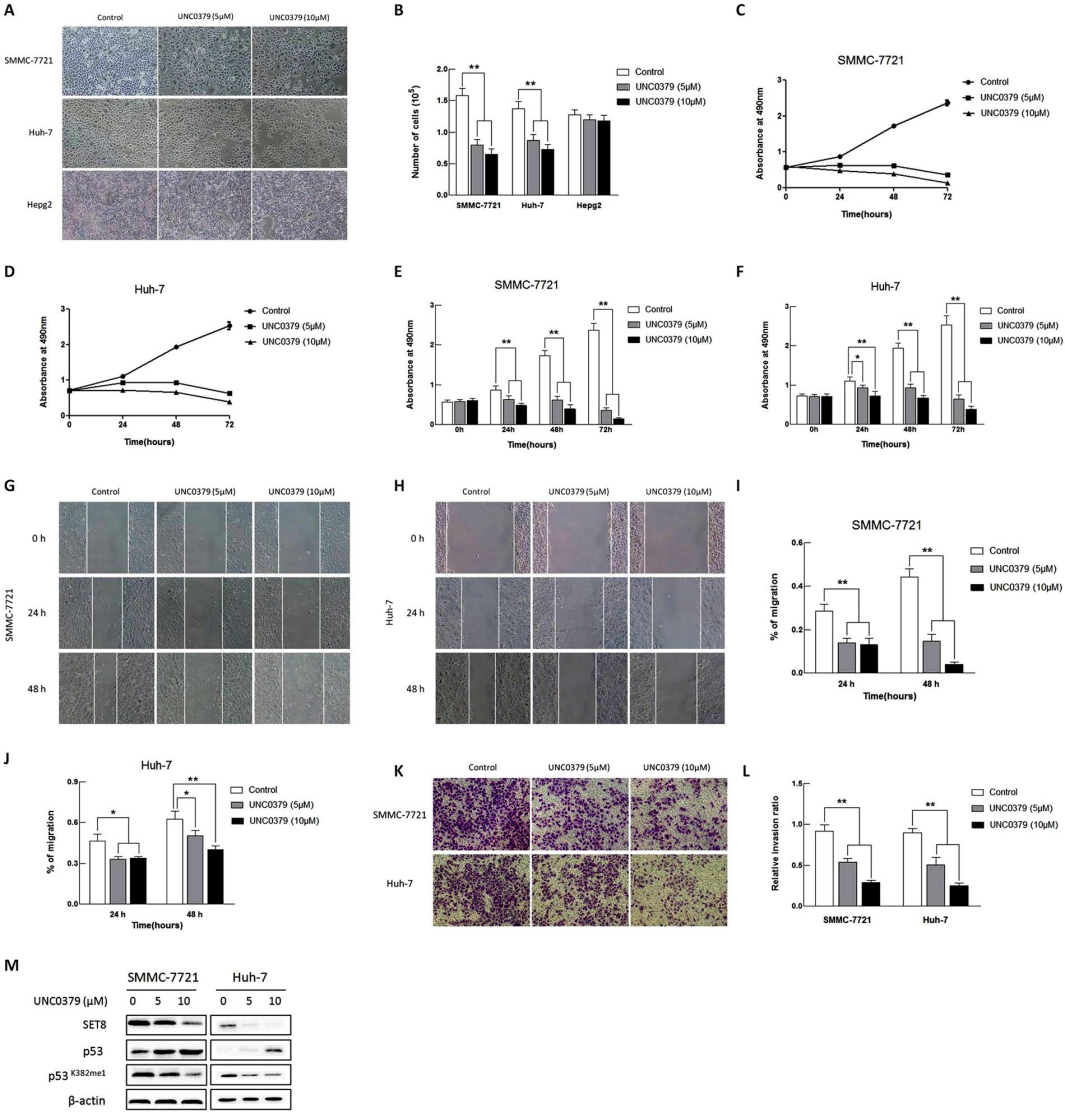
Effects of UNC0379 on proliferation, migration and invasion in HCC cells. (**A,B**) UNC0379 treatment reduces the number of HCC cells. (**C,D**) CCK-8 assay showing that treatment with 5 μM and 10 μM UNC0379 inhibits proliferation of SMMC-7721 and Huh-7 cells. (**E,F**) Quantification of results from C and D. (**G,H**) Wound healing assay showing that treatment with 5 μM and 10 μM UNC0379 suppresses migration of SMMC-7721 and Huh-7 cells. (**I,J**) Quantification of results from G and H. (**K**) Transwell assay showing that treatment with 5 μM and 10 μM UNC0379 suppresses invasion of SMMC-7721 and Huh-7 cells. (**L**) Quantification of results from K. (**M**) Western blot showing the protein levels of SET8, p53 and p53K382me1 in SMMC-7721 and Huh-7 cells treated with different concentrations of UNC0379 (5 or 10 μM) for 48 h. **p < 0.01, *p < 0.05.

UNC0379, a selective and substrate-competitive inhibitor of SET8^22^, was used to evaluate whether it mimics the effect of *SET8 siRNA* on HCC progression. SMMC-7721 and Huh-7 cells were treated with different concentrations of UNC0379 (0, 5 or 10 µM) for 72 h. Our results showed that growth of SMMC-7721 and Huh-7 cells was dramatically inhibited by UNC0379 (Fig. 3A,B). UNC0379 inhibited proliferation from 24 to 72 h (P < 0.01, Fig. 3C,D,E,F), migration (P < 0.05, Fig. 3G,H,I,J) at 24 and 48 h, and invasion (P < 0.01, Fig. 3K,L) in these two cell lines. This inhibitor also mimicked the effect of *SET8* siRNA regarding the increase in p53 levels and the decrease in p53K382mel (Fig. 3M). These data demonstrated that SET8 was an effective target for HCC prevention.

### Knockdown of SET8 increased sensitivity of SMMC-7721 cells for docetaxel

Since we showed that SET8 may be a target for HCC prevention, we examined the synergy of SET8 knockdown with chemotherapy. We assessed whether SET8 knockdown could change chemotherapy sensitivity of SMMC-7721 cells in different concentrations of chemotherapeutic agents, such as cisplatin, docetaxel, 5-Fu and etoposide. CCK-8 assay revealed that proliferation of SMMC-7721 cells was significantly inhibited in the *SET8-siRNA* group after treatment with docetaxel in all doses used (P < 0.01, Fig. 1J). These results indicated that SET8 knockdown increased chemotherapy sensitivity of SMMC-7721 cells to docetaxel.

### Gene expression profile of SET8-knockdown cells

**Figure 4:**
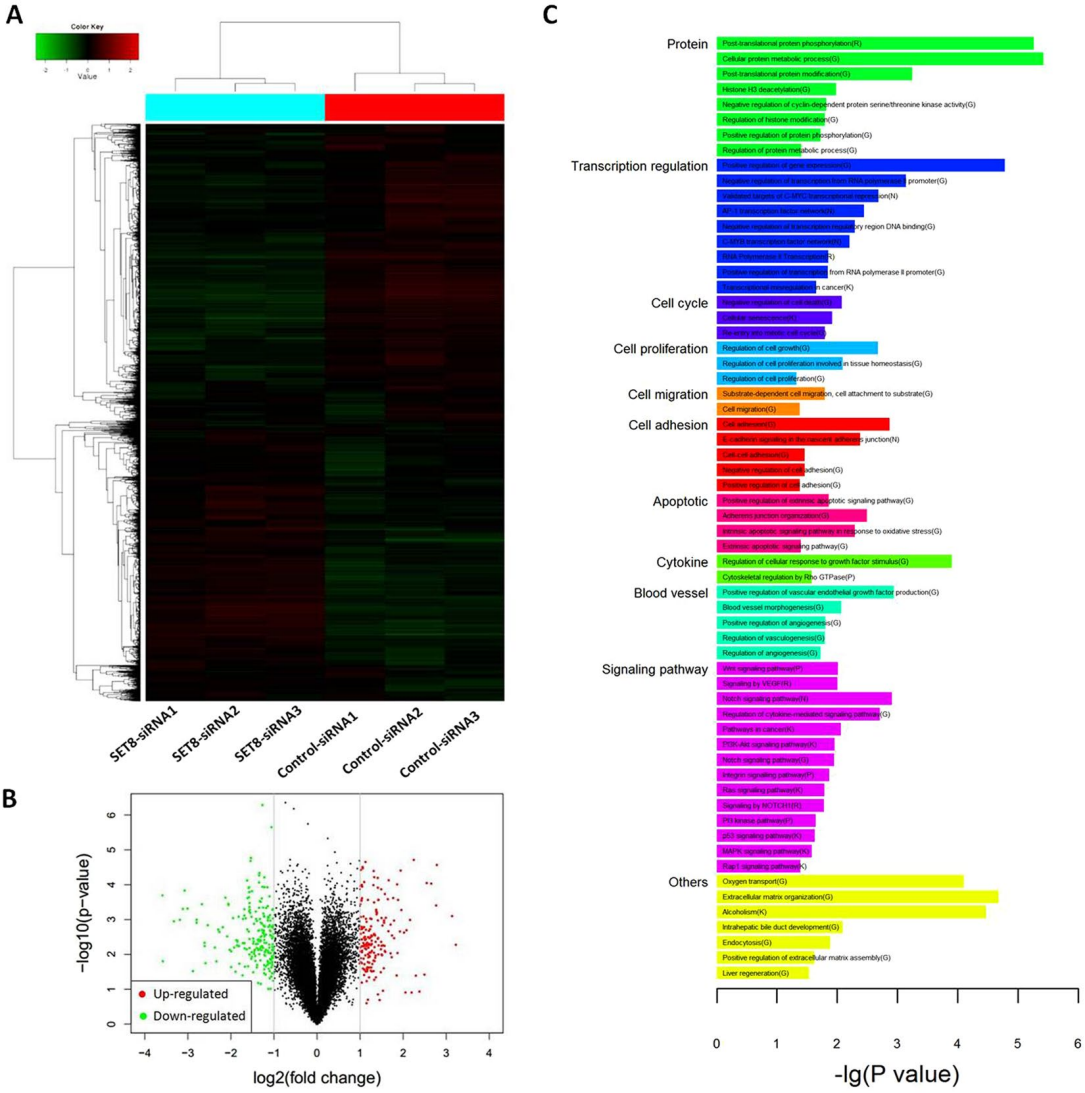
Gene expression profiling revealed the genome-wide influence of *SET8- siRNA* on SMMC-7721 cells. (**A**) Heat map analysis of differentially expressed genes in *SET8-siRNA* and control-siRNA treated cells. Up-regulated and down-regulated genes are shown in red and green, respectively. (**B**) Volcano plot showing the expression of genes in *SET8-siRNA* and control-siRNA treated cells. Red dots indicate a > 2.0-fold change in mRNA between *SET8-siRNA* and control-siRNA cells. Green dots indicate a < −2-fold change between *SET8-siRNA* and control-siRNA cells. (**C**) Reactome FI analysis with 406 genes with significantly altered expression upon SET8 knockdown. ‘G’ represents ‘GO biological process’, ‘K’ represents ‘KEGG pathway’, ‘N’ represents ‘NCI PID’, ‘R’ represents ‘Reactome’, ‘P’ represents ‘Reactome Pathway’ and ‘B’ represents ‘BioCarta’. The detailed results are shown in Supplementary Table S2.

To investigate the target genes induced by SET8 knockdown in HCC cells, we monitored the changes in mRNA levels between *SET8-siRNA* cells and control-siRNA cells with whole-genome expression microarray analysis (Fig. 4A). A total of 406 differentially expressed genes were identified, including 181 upregulated and 225 downregulated genes (fold change> 2.0, *q* < 0.05) (Fig. 4B, Supplementary Table S1). Biological network analysis for these 406 differentially expressed genes with Reactome FI showed that enrichment of biological processes related to the cell cycle, cell proliferation, cell migration, cell adhesion, apoptosis, cytokine, angiogenesis and the enriched signalling pathways, namely p53 signalling pathway^23^, Wnt signalling pathway^24^ and VEGF signalling pathway^25^, were associated with SET8 knockdown (Fig. 4C, Supplementary Table S2). These data demonstrated that SET8 modified the progression of HCC by modulating these signalling pathways.

## Discussion

In the present study, we evaluated the function of SET8 in HCC development. We showed that SET8 knockdown inhibited proliferation of HCC cells both *in vitro* and *in vivo*. In addition, SET8 knockdown inhibited migration and invasion of HCC cells. These results are in line with previous studies that highlighted the role of SET8 in progression and metastasis of a variety of tumours, such as papillary thyroid cancer, oesophageal squamous cell carcinoma and renal cell carcinoma^26–28^. Consistent with these data, SET8 overexpression and UNC0379 treatment also proved that SET8 is an oncogene that favours HCC development. The fact that SET8 knockdown increased sensitivity of SMMC-7721 cells to docetaxel treatment implied the potential for SET8 to be used as a treatment target and biomarker for treatment efficiency. Docetaxel is a member of the taxane family that is known to disrupt microtubule formation during cell division. This drug has displayed antiproliferative activity through induction of HCC cell death in a previous study^29^. The potential prevention mechanism of docetaxel and its interaction with SET8 in HCC progression require further study in a larger sample size and in animal models.

A total of 406 genes were significantly altered in SET8 knockdown HCC cells, based on microarray analysis. Reactome FI analysis showed that genes altered in many biological processes referring to the cell cycle, cell proliferation, cell migration, cell adhesion, apoptosis, cytokine, angiogenesis, the Wnt signalling pathway,VEGF signalling pathway and p53 signalling pathway upon SET8 knockdown, which implied that these processes may be modified by SET8 alteration.

The Wnt signalling pathway is essential for a number of cellular functions including cell proliferation, migration and invasion^30^. SET8 knockdown inhibited proliferation, migration and invasion of renal carcinoma 786-O cells, potentially through Wnt/β-catenin signalling^28^. Our results also demonstrated that the Wnt signalling pathway may be responsible for modulating cellular functions upon SET8 knockdown. VEGF is a major driver of angiogenesis in carcinogenesis and cancer progression that correlates with vascular and portal vein invasion and influences HCC outcome^31^. In addition, the novel anti-VEGF humanised monoclonal antibody BD0801 significantly inhibited proliferation of SMMC-7721, and caused cell cycle arrest at the G1 phase^25^. Taken together, all these signalling pathways may modify the outcome of HCC upon SET8 knockdown. Functional analysis of candidate genes should be performed in further studies.

SET8 also catalyses monomethylation of H4K20me1 and of non-histone proteins, such as p53, TWIST, Wnt, and PCNA. Accumulating evidence has shown that SET8 is crucial for gene transcription regulation during tumour formation and progression^6,7,9^. It has been reported that SET8 modulates p53 activity through methylation at K382 and knockdown of SET8 resulted in p53 reduction in papillary thyroid cancer cells^26^. We did find that SET8 modulated p53 expression through methylation of K382 in HCC cells. SET8 may influence HCC development through methylation of p53 and subsequent downstream signal changes. Additionally, SET8 induced epithelial-mesenchymal transition (EMT) through interaction with TWIST, thereby enhancing cell invasion in breast cancer^12^. Moreover, SET8 interacts directly with the DNA replication factor PCNA and shows specific effect at the origins of replication^32^. Taken together, SET8 could play an oncogenic role by facilitating tumorigenesis.

In summary, our study suggests that SET8 may modify HCC development and progression by influencing proliferation, migration and invasion through modulation of a number of genes. Therefore, SET8 may be a potential new therapeutic target for HCC treatment.

## Supplementary information

Supplementary table legends.

Dataset 1.

Dataset 2.

## Data Availability

All data generated or analysis during this study are included in this published article (and its Supplementary Information files).
