# Supplementary material for: Downregulation of histone methyltransferase SET8 inhibits progression of hepatocellular carcinoma
Source: Sci Rep. 2020 Mar 11;10:4490. doi: 10.1038/s41598-020-61402-7 (PMC7066161; doi:10.1038/s41598-020-61402-7)
Supplement: Supplementary file 1 — Supplementary table legends. [file 41598_2020_61402_MOESM1_ESM.docx]

Downregulation of histone methyltransferase *SET8* inhibits progression of hepatocellular carcinoma

Jianhua Wu^1,#^, Kuangyuan Qiao^4,#^, Yanming Du^2^, Xiaoyun Zhang^2^, Haichao Cheng^3^, Li Peng^3,^*, Zhanjun Guo^2,^*

^1^Animal center, ^2^Departments of Immunology and Rheumatology, ^3^Department of Hepatobiliary Surgery，The Fourth Hospital of Hebei Medical University, Shijiazhuang, P.R. China.

^4^Basic Medical College, Hebei Medical University, Shijiazhuang, P.R. China.

^#^These authors contribute equally to this work.

Running title: SET8 inhibits HCC progression.

*To whom correspondence should be addressed:

Li Peng, M.D. and Ph.D

Department of Hepatobiliary Surgery, The Fourth Hospital of Hebei Medical University, 12 Jiankang Road, Shijiazhuang 050011, P.R. China.

E-mail: pengli72@sina.com

and

Zhanjun Guo, M.D. and Ph.D

Departments of Immunology and Rheumatology, The Fourth Hospital of Hebei Medical University, 12 Jiankang Road, Shijiazhuang 050011, P.R. China.

Tel: + 86 311 8609 5342. Fax: + 86 311 8609 5237.

E-mail: [zjguo5886@aliyun.com](mailto:zjguo5886@aliyun.com)

Supplementary table legends

Supplementary Table S1. Genes with significantly altered expression following SET8 knockdown in SMMC-7721 cells.

Supplementary Table S2. Biological network analysis of genes with significantly altered expression after SET8 knockdown in SMMC-7721 cells.
